# *In vitro* Implementation of Photopolymerizable Hydrogels as a Potential Treatment of Intracranial Aneurysms

**DOI:** 10.3389/fbioe.2020.00261

**Published:** 2020-04-03

**Authors:** Oriane Poupart, Andreas Schmocker, Riccardo Conti, Christophe Moser, Katja M. Nuss, Hansjörg Grützmacher, Pascal J. Mosimann, Dominique P. Pioletti

**Affiliations:** ^1^Laboratory of Biomechanical Orthopedics, EPFL, Lausanne, Switzerland; ^2^Laboratory of Applied Photonics Devices, EPFL, Lausanne, Switzerland; ^3^Department of Chemistry and Applied Biosciences, ETH, Zurich, Switzerland; ^4^Institute of Diagnostic and Interventional Neuroradiology, Inselspital, Bern University Hospital, Bern, Switzerland; ^5^Musculoskeletal Research Unit, Department of Molecular Mechanisms of Disease, Vetsuisse Faculty, University of Zurich, Zurich, Switzerland

**Keywords:** intracranial aneurysms (IA), injectable hydrogels, *in situ* photopolymerization, light-conducting microcatheter, polyethylene glycol dimethacrylate

## Abstract

Intracranial aneurysms are increasingly being treated with endovascular therapy, namely coil embolization. Despite being minimally invasive, partial occlusion and recurrence are more frequent compared to open surgical clipping. Therefore, an alternative treatment is needed, ideally combining minimal invasiveness and long-term efficiency. Herein, we propose such an alternative treatment based on an injectable, radiopaque and photopolymerizable polyethylene glycol dimethacrylate hydrogel. The rheological measurements demonstrated a viscosity of 4.86 ± 1.70 mPa.s, which was significantly lower than contrast agent currently used in endovascular treatment (*p* = 0.42), allowing the hydrogel to be injected through 430 μm inner diameter microcatheters. Photorheology revealed fast hydrogel solidification in 8 min due to the use of a new visible photoinitiator. The addition of an iodinated contrast agent in the precursor contributed to the visibility of the precursor injection under fluoroscopy. Using a customized light-conducting microcatheter and illumination module, the hydrogel was implanted in an *in vitro* silicone aneurysm model. Specifically, *in situ* fast and controllable injection and photopolymerization of the developed hydrogel is shown to be feasible in this work. Finally, the precursor and the polymerized hydrogel exhibit no toxicity for the endothelial cells. Photopolymerizable hydrogels are expected to be promising candidates for future intracranial aneurysm treatments.

## Introduction

Intracranial aneurysms (IAs) are saccular dilatations or outpouchings of brain arteries that are prone to rupture, causing death or severe morbidity in over half of the patients (Medical Advisory Secretariat, 2006). While minimally invasive endovascular therapy has many advantages and is increasingly being performed, the risk of IA recurrence remains higher compared to surgical clipping (Molyneux et al., 2015; Smith et al., 2015; Hulsbergen et al., 2019). Adjunctive devices such as flow diverting stents may decrease this risk by promoting endothelial growth at the neck of the IA (Ravindran et al., 2019) but currently require dual anti-platelet medication to avoid parent artery thrombosis, thereby increasing the risk of bleeding. Device compaction (Abdihalim et al., 2014), inflammation (Tulamo et al., 2010) and intra-saccular clot remodeling (Marbacher et al., 2019) appear to play a decisive role in healing and recurrence. Therefore, a liquid agent capable of (i) selectively filling the entire aneurysm, (ii) reversing the chronic inflammatory wall changes, (iii) preventing thrombosis, and (iv) attracting endothelial cells could eliminate the current drawbacks of endovascular therapy.

Using liquid embolic agents to treat aneurysms is not a new concept (Brassel and Meila, 2015). They allow the whole volume of aneurysms of any shape or size to be filled. The main limitation lies in their self-solidifying properties once in contact with blood, risk of spillage and downstream migration that may cause arterial occlusion and ischemic stroke. The risk of microcatheter entrapment within the solid agent, particularly with fast polymerizing substances such as acrylic glues, is also significant (Walcott et al., 2011). While non-adhesive ethyl-vinyl-alcohol (EVOH) copolymer agents precipitate over several minutes (Kilani et al., 2015), the dimethyl sulfoxide (DMSO) solvent they contain can induce vasospasm and angionecrosis depending on the concentration and volume injected (Takeda et al., 2015; Onyx^®^ Liquid Embolic System) which is especially harmful if left to stagnate in such a restricted space as an aneurysm. Moreover, leakage and solidification in the parent artery remains a serious potential complication, even with balloon assistance.

Injectable *in situ* forming hydrogels have raised considerable interest for biomedical applications (Dimatteo et al., 2018; Li et al., 2018; Zhang et al., 2019), including as potential novel embolic agents for aneurysms or transcatheter embolization. The liquid precursors of these hydrogel-based embolic agents have been triggered to solidify *in situ* by different stimuli, such as temperature (Takao et al., 2006; Zhao et al., 2013; Shi et al., 2016), pH (Nguyen et al., 2016), chemical reaction (Bearat et al., 2013; Weng et al., 2013; Zhou et al., 2016) or light irradiation (Barnett et al., 2009; Ishikawa et al., 2012). Compared to the others stimuli, light-induced photopolymerization allows better spatial and temporal control of the curing (Doran et al., 2015; Corrigan et al., 2019). This control is essential to avoid any premature gelation or later dilution that could occur with temperature-sensitive, pH-sensitive and chemically crosslinked types. To our knowledge, only a few studies have developed photopolymerizable hydrogels for IA treatment. Ishikawa et al. (2012) have reported *ex situ* photopolymerization of hydrogels in an aneurysm model whereas Barnett et al. (2009) described *in situ* photopolymerization of hydrogels in a saccular aneurysm model using 2.8 French-microcatheter (700 μm inner diameter). To demonstrate the feasibility of *in vitro* implementation of photopolymerizable hydrogels, many requirements need to be taken into consideration. First, the hydrogel needs to be biocompatible and safe to be injected in its liquid state in the neuro- and cardiovascular systems, as a fraction of the precursor is bound to leak into the blood flow, even under balloon assistance. Second, it has to exhibit low viscosity to be injectable through a microcatheter with an inner diameter of 430 μm, such as those used for coil delivery in current clinical practice. Moreover, the hydrogel needs to be radiopaque to be visible under fluoroscopy. Furthermore, despite evidence that a balloon can remain inflated and block the flow in the parent artery for as long as 18 consecutive minutes (Spiotta et al., 2011), photopolymerization time should remain as short as possible, 10 min at most, ideally a few seconds, to diminish the risk of ischemic stroke and vessel injury. Finally, use of light in the visible spectrum to induce photopolymerization should be preferred over ultraviolet emission to avoid possible damage to encapsulated cells, host tissues or DNA (Sinha and Häder, 2002; Fedorovich et al., 2009) and allow deeper light penetration within a highly absorbing medium such as blood.

**FIGURE 1:**
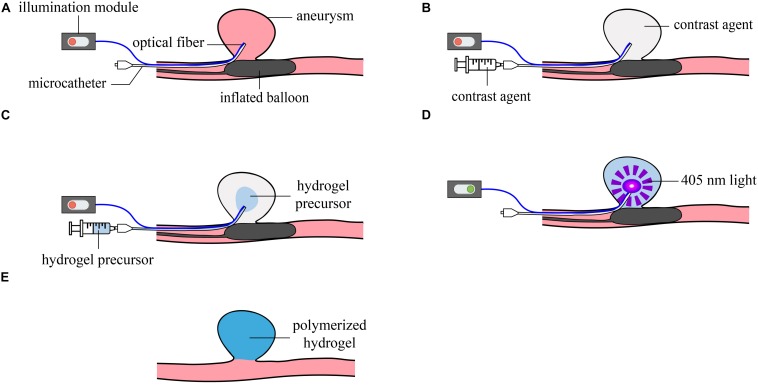
Schematic illustration of the different steps of the new treatment concept. **(A)** Positioning of the light-conducting microcatheter in the aneurysm and inflation of the balloon at the neck; **(B)** Rinsing of the aneurysm with contrast agent; **(C)** Injection of the hydrogel precursor; **(D)** 405 nm illumination and photopolymerization of the hydrogel; **(E)** Removal of the microcatheter and balloon.

Photopolymerizable polyethylene glycol dimethacrylate (PEGDMA) hydrogels have been widely used for tissue engineering (Burke et al., 2019). Indeed, PEGDMA hydrogels present many advantages such as tunable mechanical properties, ease of chemical conjugation and biocompatibility. Herein, we describe the use of a radiopaque photopolymerizable hydrogel obtained from PEGDMA mixed with clinically approved iodinated contrast medium and a next-generation visible light-sensitive and water-soluble photoinitiator, known as poly(ethylene glycol) substituted bis(acyl)phosphane oxides (PEG-BAPO) (Wang J. et al., 2018). To address the challenge of inserting a photoactivable hydrogel into an IA, we also present the development of a light-conducting microcatheter (inner lumen of 430 μm) combined with an illumination module to guide visible light to the aneurysm. We hereby report a proof-of-concept study to treat IAs with photopolymerizable hydrogels under fluoroscopy in a realistic flow model (Figure 1), as well as a preliminary biocompatibility evaluation of the hydrogel precursor.

## Materials and Methods

### Materials

**FIGURE 2:**
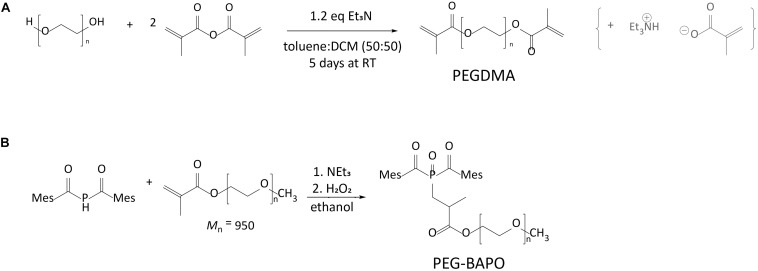
**(A)** Synthesis of PEGDMA polymer. **(B)** Synthesis of PEG-BAPO photoinitiator.

All the chemicals were purchased from Sigma Aldrich (Merck, Switzerland). The reactions were performed under inert atmosphere of dry Argon using Schlenk techniques. Dry solvents were drawn from a commercial solvent drying system (Innovative Technology PURESOLV). The reaction flasks were dried in an oven overnight at 120°C before use. PEGDMA 6 kDa was prepared from poly(ethylene glycol) (PEG, M_n_ = 6000 g/mol) using a modified procedure from the previously reported one (Lin-Gibson et al., 2004), as shown in Figure 2A. The polymer (50 g) was loaded in a three necked 500 ml round bottomed flask equipped with an adapter for the Schlenk line and a Dean Stark condenser. The polymer was dissolved in 200 ml of toluene. The mixture was stirred at 600 rpm and warmed to 160°C. The mixture was refluxed for 1 h and every 15 min an aliquot of 25 ml of solvent was removed using the Dean-Stark condenser. After 100 ml of solvent were removed, the mixture was cooled down to room temperature. Dry dichloromethane (100 ml) was added to the mixture followed by Et_3_N (distilled from CaCl_2_, stored in a Schlenk under Argon, 1.1 eq. for every OH) and methacrylic anhydride (distilled from CaCl_2_, stored in a Schlenk under Argon, 1.5 eq. for every OH). The mixture was protected from light and stirred at room temperature for 5 days. Subsequently, it was purified by filtration over a neutral alumina plug. The polymer was coagulated into diethyl ether and dried under high vacuum. The white powder was obtained in quantitative yield and characterized as the desired product via ^1^H-NMR. The degree of functionalization was evaluated by integration of the ^1^H-NMR resonances. The chemical structure of PEGDMA was confirmed using infrared spectroscopy (FT-IR, Bruker Tensor 27, Germany) in the infrared radiation range of 4000-400 cm^–1^ with a resolution of 4 cm^–1^ and 256 scans.

The iodine-based contrast medium Accupaque (350 mg/ml, GE Healthcare, Chicago, IL, United States) was provided by the Bern University Hospital. Intralipid 20%, an intravenous fat emulsion, was purchased from Sigma Aldrich (Merck, Switzerland).

The PEG-BAPO photoinitiator (PI) was synthetized by phospha-Michael addition of bis(mesitoyl)phosphane (BAP-H) to poly(ethylene glycol) methyl ether methacrylate (PEGMEM) followed by oxidation with aqueous H_2_O_2_, as previously reported (Wang J. et al., 2018). The synthesis of PEG-BAPO is shown in Figure 2B.

### Hydrogel Precursor Preparation

The hydrogel precursor was prepared by dissolving PEGDMA (10 wt%) in equivalent weight concentrations of phosphate buffered saline (PBS) and Accupaque350. Since PEG-BAPO PI was shown to be more efficient (higher curing rate) than other commercially available water-soluble photoinitiators like Irgacure2959 (Wang J. et al., 2018), we decided to add this (0.1 wt%) to the formulation. This hydrogel precursor formulation was homogenized by Vortex for 5 min, degassed using a vacuum pump and then passed through a 0.22 μm syringe-filter (Rotilabo, Carl Roth, GmbH) for sterilization. Finally, intralipids (5 wt%) were added to the hydrogel precursor to enhance light-scattering and increase the polymerized volume during subsequent illumination (Schmocker et al., 2014).

### Injectability of the Hydrogel Precursor

The rheological properties of the hydrogel precursor were assessed with a modular compact rheometer 102 (Anton Paar, Peseux, Switzerland) by an oscillatory time sweep with a strain oscillation of 5% at a frequency of 1 Hz applied during 5 min. The dynamic viscosity was calculated as the average of the real part of the complex viscosity. The viscosity of the precursor was then compared with two reference samples, namely water and Accupaque350.

### Radiopacity of Precursor and Hydrogels

Using pulsed fluoroscopy (7.5-10 p/s) and serial angiography snapshots (70 kV, 23 mA) on a HDR flat detector monoplane system (154 μm pixel size, 16-bit digitization depth, Siemens Artis Zee, Erlangen, Germany), the radiopacity of 2 mm-thick samples of liquid precursor was assessed and compared to an equal volume of solid hydrogel at t_0_ (immediately after polymerization) and after incubation in PBS for 36 h. The results were compared to pure Accupaque350 and to a single lumen occlusion balloon (Scopernic15, Balt, Montmorency, France) containing two radiopaque markers.

### Photopolymerization

For photorheology, the light from a 405 nm wavelength light laser diode was coupled to the modular compact rheometer 102 and a parallel 25 mm diameter disk configuration was used. 200 μl samples of the hydrogel precursor were tested directly after hydrogel preparation. A strain oscillation of 5% at a frequency of 10 Hz was applied and the temperature fixed at 37°C. Samples of 300 μm thickness were illuminated with a light intensity of 15 mW/cm^2^ at 405 nm. The evolution of the storage modulus G′ was recorded during 405 nm irradiation. The polymerization was considered complete when G′ reached a plateau level. The time required to reach 95% of the plateau level of G′ was defined as polymerization time, noted t_95_, as previously reported (Khoushabi et al., 2015).

### *In vitro* Aneurysm Model

**FIGURE 3:**
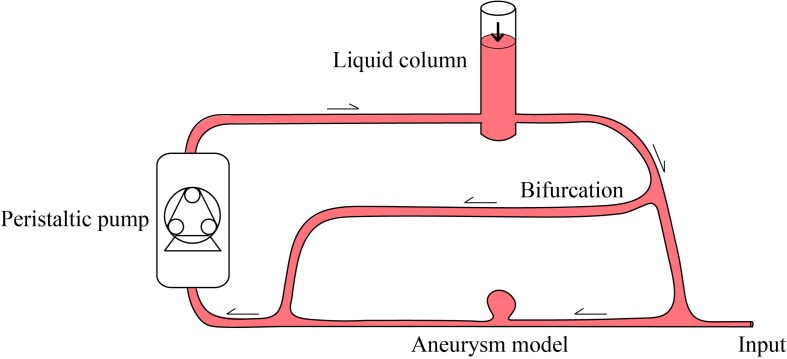
Schematic illustration of the *in vitro* setup to evaluate the feasibility of the implantation. The peristaltic pump induced a pulsatile flow of 220 ml/min at 1 Hz. The height of the liquid column ensured a constant pressure of 12 kPa to the system. A parallel bifurcation channel, representing collateral shunting or outflow in the head and neck during balloon inflation at the aneurysm neck was added to dissipate over-pressurization and avoid bursting or displacing the balloon when inflated. The input represents access to the internal carotid artery through which a 9 French introducer sheath and an 8 French guiding catheter were placed, followed by navigation of the light-conducting microcatheter and balloon to the aneurysm site.

The feasibility of hydrogel injection, photopolymerization and implantation was evaluated in an *in vitro* aneurysm model shown in Figure 3. Since approximately 30% of IAs develop on the internal carotid artery, where flow rate and turbulence in the aneurysm are higher compared to more frequent aneurysm locations such as the anterior communicating or middle cerebral artery (Bonneville et al., 2006), we decided to model a paraophthalmic IA to simulate the worst hemodynamic conditions. The setup consisted of a wide necked, 5 × 6 mm left carotid ophthalmic aneurysm made of soft silicone (Elastrat, Geneva, Switzerland), connected to a peristaltic pump (Masterflex 7523-37, ColeParmer, Vernon Hills, IL, United States) and head pump (Masterflex 7036-30, ColeParmer, Vernon Hills, IL, United States) using 9.7 mm inner diameter silicone tubing (Masterflex 96410-36, ColeParmer, Vernon Hills, IL, United States). The pump parameters were set to 1 Hz and 220 ml/min to mimic human internal carotid artery flow rate (Enzmann et al., 1994). Additionally, a vertical 1.2 m liquid column inducing a constant pressure of 12 kPa (90 mmHg) to mimic intracranial arterial pressure conditions (91.2 ± 9.6 mmHg) (Netlyukh et al., 2015) was implemented. The system was filled with a blood substitute composed of 36 vol% glycerol in water and PicroSirius Red dye (0.134 wt%) to match blood viscosity (Lim et al., 2001) and light absorbance, respectively. The dye concentration was determined using the Beer-Lambert law (Supplementary Figure S1).

### *In vitro* Proof-of-Concept of the Implementation

**FIGURE 4:**
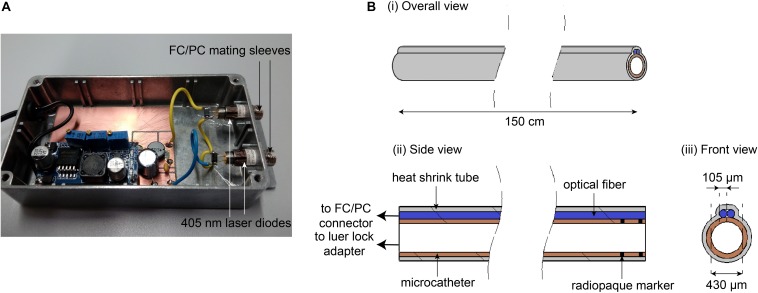
**(A)** Illumination module to which the optical fibers are connected. **(B)** Schematic of the light-conducting microcatheter from different views (i) overall, (ii) side, and (iii) front view.

In order to bring light to the aneurysm to photopolymerize the hydrogel precursor *in situ*, two optical glass fibers (105 μm, 0.22 NA, 1.5 m, ThorLabs, Newton, NJ, United States) were wrapped with heat shrunk hydrophilic tube (Vesta, Lubrizol Company, Corona, CA, United States) along the outer surface of a microcatheter typically used for IA coiling (Headway 17, MicroVention, Tustin, CA, United States). The tip of the optical fibers was fixed to match the distal marker of the microcatheter and connected proximally to an illumination module composed of 405 nm, 200 mW light power laser diodes (Fibotec Fiberoptics GmbH, Meiningen, Germany), as shown in Figure 4. First, a 9F introducer sheath (Radifocus Introducer II, Terumo, Tokyo, Japan) and 8F guide catheter (Mach1, Boston Scientific, Marlborough, MA, United States) were inserted in the input tubing. Under fluoroscopic guidance, the light-conducting microcatheter was placed in the aneurysm sac followed by the placement of an inflatable balloon (Scopernic15, Balt, Montmorency, France) at the neck of the aneurysm. The balloon was inflated, with a solution of Accupaque350 for radiopacity and black ink to prevent light from polymerizing leaked material in the parent artery. The blood substitute was flushed out from the aneurysm cavity with a contrast agent injected through the microcatheter under submaximal balloon inflation, in order to minimize light absorption caused by red dye and therefore maximize photopolymerization efficiency. The hydrogel precursor was injected under blank roadmap fluoroscopy using a dedicated 3 ml syringe wrapped in aluminum foil (to avoid premature polymerization caused by ambient light) until the contents of the aneurysm were completely replaced and started to leak out downstream. After sealing the neck with maximal balloon inflation, the precursor was illuminated by the optical fibers for 8 min. Following photopolymerization, the microcatheter was removed under balloon inflation to avoid fragmentation followed by progressive balloon deflation and removal.

### Cell Viability Assay

The cytotoxicity of the precursor was investigated by cell viability assay. Human umbilical vein endothelial cells (HUVEC) were cultured on a 96-well plate with 10,000 cells/well and 100 μl of culture medium in each well. HUVEC were incubated for 48 h and evaluated for cell attachment and confluence. The culture medium was aspirated and 100 μl of different conditions of precursors were placed in contact with cells during 1 week. Constant concentrations of 0.1, 0.5, 1, 2.5, 5, and 10 vol% were tested. Three rounds of serial dilutions of the precursor were also performed. For each of the dilution rounds, pure precursor was placed in contact with HUVEC for 10 min, aiming to simulate the contact of HUVEC with the precursor during the injection and photopolymerization, 10 min being the maximum polymerization time required for aneurysm application. Then, the precursor was diluted at three different speeds, as shown in Supplementary Figure S2, corresponding to the potential dilution of the precursor into the blood stream during leakage in the parent artery. To evaluate the cell viability after several time points (24 h, 72 h, and 168 h), the medium was aspirated and each well was filled with 100 μl of PBS with 10 vol% PrestoBlue assay (A13261, Life Technologies). The culture medium was replaced every 3 days. The fluorescence at 595 nm was measured by a microplate reader (Wallac 1420 Victor2, PerkinElmer) after 30 min of incubation. Wells without precursor were considered as positive controls.

To examine the possible toxicity of the solid, polymerized hydrogels, HUVEC were cultured on a 48-well plate with 100,000 cells/well and 1 ml of culture medium in each well. The cells were incubated during 24 h for cell attachment and confluence. Cell strainers (70 μm pluriStrainers, pluriSelect) were laid on the top of the wells and hydrogel samples were placed in the strainers. Since unreacted particles, mainly the photoinitiator, can be released into the blood stream during hydrogel immersion and have a negative impact on cell viability, we assessed their impact with a PrestoBlue assay immediately after polymerization and after 1 day of incubation in PBS. The culture medium was replaced every 3 days.

### Platelet Aggregation Assay

Platelet aggregation, for different concentrations of precursor ranging from 0.1 to 10 vol%, was determined as previously described (Lima et al., 2019). Briefly, healthy human blood samples were centrifuged for 15 min, 200 g at room temperature (RT) and platelet-rich plasma (PRP) was collected. Blood samples were centrifuged again for 15 min, 500 g at RT and platelet-poor plasma (PPP) was collected. Twenty μl of different concentrations of precursor were placed in a 384-well plate (Greiner Bio One, ref 781903) and 20 μl of PRP was added in each well. The absorbance at 595 nm was measured after 15 min incubation, 1200 RPM at RT and platelet aggregation was determined by normalization with Tyrode buffer (0% aggregation) and PPP (100% aggregation). Collagen I (Mölab, ref 0203009) at 5 μg/ml was also used as positive control.

### Statistical Analysis

Each experiment was performed in triplicate or more and each statistical evaluation was done with MATLAB (Mathworks, Natick, MA, United States). All data are expressed as mean ± standard deviation. One-way analysis of variance (ANOVA) was used for comparison. *P* < 0.05 was considered as a significant result (denoted as ^∗^) and *p* > 0.05 non-significant (denoted ns). *P* < 0.01 was denoted as ^∗∗^ and *p* < 0.001 as ^∗∗∗^.

## Results

### Characterization of PEGDMA Polymer

**FIGURE 5:**
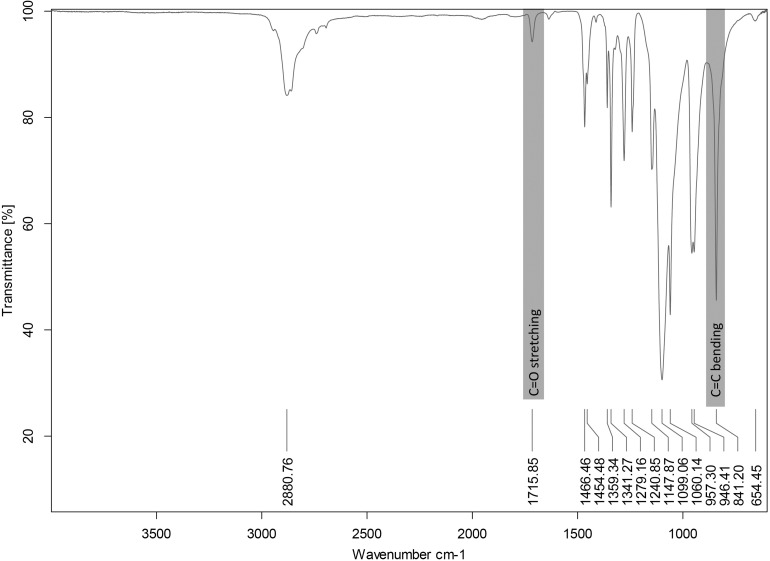
FT-IR spectrum of PEGDMA polymer.

The degree of functionalization of PEGDMA was found by ^1^H-NMR integrals to be over 90%. The functionalization of the PEG with methyl methacrylate ending units was confirmed by the FT-IR spectra, shown in Figure 5, recorded on the purified product. The signal arising from the C = O stretching methyl methacrylate substituent was visible at 1715.85 cm^–1^. A peak at 841.20 cm^–1^ was also observed, corresponding to the C = C bending. Thus, the methacrylation functionalization of the PEG polymer was confirmed by FT-IR.

### Injectability of Hydrogels Precursors

**FIGURE 6:**
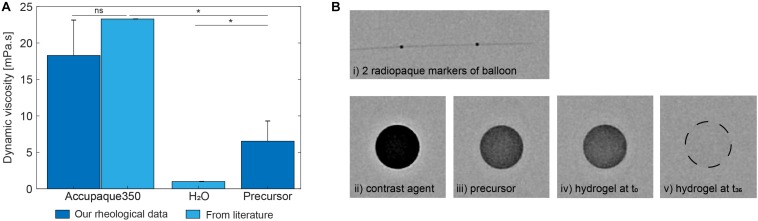
**(A)** Dynamic viscosities for Accupaque350, water, and hydrogel precursor by rheology measurements (*n* = 3). **(B)** Radiography snapshot of (i) a deflated balloon with its two proximal and distal radiopaque markers, (ii) pure Accupaque350, (iii) hydrogel precursor, (iv) polymerized hydrogel immediately after photopolymerization and (v) polymerized hydrogel after 36 h-immersion in PBS.

The dynamic viscosity at room temperature (23°C) of the precursor and the contrast agent Accupaque350 were 6.53 ± 2.77 and 18.30 ± 4.85 mPa.s, respectively (Figure 6A). The water viscosity of 1 mPa.s from literature was used as reference. Comparatively, precursor viscosity was significantly lower than Accupaque350 value (*p* = 0.022) and significantly higher than water (*p* = 0.026). In addition, viscosities of other liquid embolic agents, such as Onyx or PHIL, currently used in endovascular therapy, range from 16 to 72 mPa.s (Vollherbst et al., 2017). Therefore, our precursor should be injectable through microcatheters.

### Radiopacity of Hydrogels Precursors

The 2 mm-thick samples observed under fluoroscopy are shown in Figure 6B. The radiopacity was compared with pure Accupaque350 and the two radiopaque markers of a single lumen occlusion balloon. The precursor and polymerized hydrogel were clearly visible under fluoroscopy, although less radiopaque than pure contrast agent. As expected, the iodinated content of the hydrogel was no longer visible after 36 h of incubation in PBS.

### Photopolymerization of Hydrogels

**FIGURE 7:**
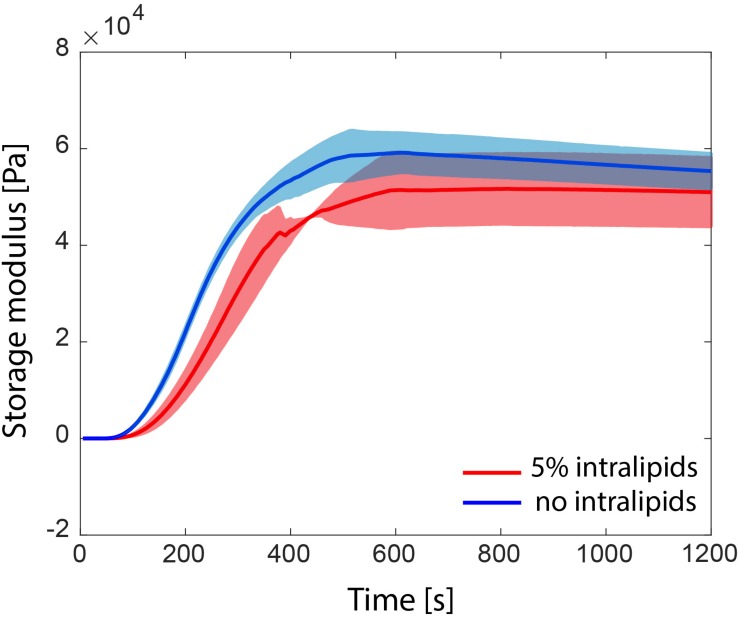
Storage modulus G’ evolution during the photopolymerization of PEGDMA hydrogels with and without intralipids (*n* = 3).

Photorheology measurements showed that the hydrogel precursor completely polymerized after 8 min 15 s ± 1 min 36 s under a 405 nm illumination at 15 mW/cm^2^, as shown in Figure 7. When intralipids were added (5 wt%), the photopolymerization time *t*_95_ was non-significantly reduced to 7 min + 38 s ± 1 min 16 s (*p* = 0.6318). In addition, the G′ plateau was measured to be 59.48 ± 4.70 kPa and 51.43 ± 7.53 kPa, without and with 5% intralipids respectively (*p* = 0.1914).

### *In vitro* Proof-of-Concept of Implementation

**FIGURE 8:**
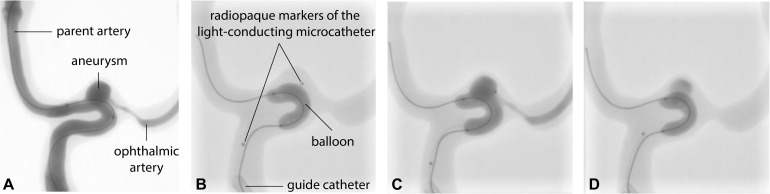
Subtracted **(A)** and **(B–D)** unsubtracted fluoroscopic images showing **(A)** contrast agent injection into the model to visualize the dimensions of the parent artery, aneurysm and ophthalmic artery; **(B)** guide catheter, inflated balloon (slightly prolapsing in the aneurysm) and light-conducting microcatheter in place; **(C)** injection of the hydrogel precursor through the microcatheter and **(D)** polymerized hydrogel after 8 min of 405 nm illumination and removal of the light-conducting microcatheter.

**FIGURE 9:**
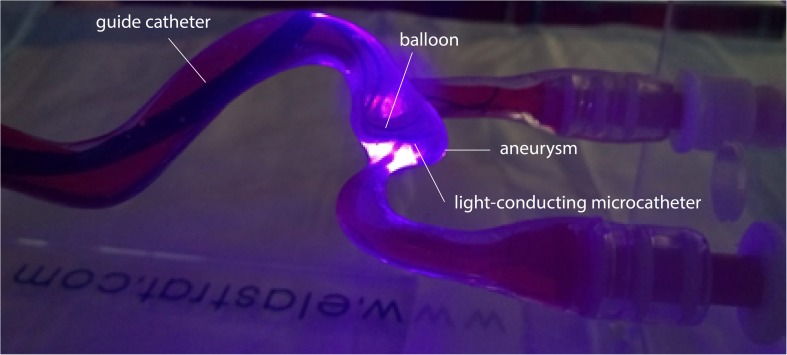
Optical fiber *in situ* illumination through the microcatheter in the aneurysm.

The light-conducting microcatheter (Figure 5B) was subjectively stiffer than the standard clinical microcatheter, due to the heat shrink tube wrapping around the glass optical fibers along the outer surface but this did not affect the navigation inside the silicone model and also ensured mechanical protection of the fibers. After positioning the balloon at the aneurysm neck in the parent vessel, the distal tip of the microcatheter (distal radiopaque marker) was placed inside the aneurysm within a few minutes, as shown in Figure 8A. Figure 9 shows the optical fiber illumination *in situ*.

Fluoroscopically controlled balloon inflation was easily achieved (Figure 8B) followed by contrast agent injection to rinse the blood substitute volume out of the aneurysm cavity. Subsequently, the hydrogel precursor was injected through the microcatheter with a similar resistance to a regular contrast injection, according to the experienced neuro-interventionalist performing the injection (PJM) and depicted in Figure 8C. The aneurysm was considered to be completely filled by the hydrogel precursor after all the contrast agent was replaced under blank map fluoroscopy (initially no contrast visible followed by negative contrast [white aspect] during contrast agent removal and finally positive contrast [black aspect] as the precursor filled the cavity). Direct visual inspection was used to confirm the complete filling of the aneurysm. A small residual inflow from the parent artery in the aneurysm was observed, despite maximal balloon inflation at the junction between the balloon and the microcatheter. This inflow was probably due to the rigidity and low compliance of the silicone model, a feature not generally observed in physiological conditions, unless the balloon leaks or is not sufficiently inflated. To match physiological conditions, the pump parameters were adapted until flow was almost stopped (25 ml/min) while the balloon was inflated. Despite this corrective measure, we observed some leakage of the precursor in the parent vessel and ophthalmic artery. The optical fibers connected to the illumination module were then used to illuminate the aneurysm with 25 mW/cm^2^ of light at 405 nm. After 8 min of continuous *in situ* illumination of the hydrogel precursor (in a darkened room with no ambient light), the microcatheter was removed under fluoroscopy without fragmenting the hydrogel, suggesting minimal adhesion to the microcatheter.

**FIGURE 10:**
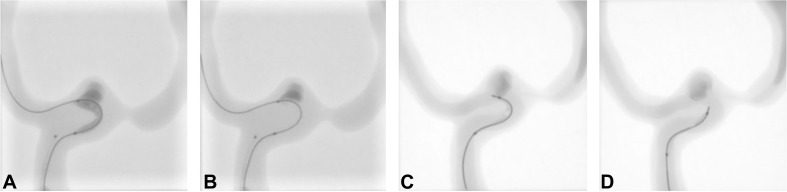
Unsubtracted fluoroscopic images of **(A)** deflation and **(B–D)** progressive removal of the balloon. Note that radiopacity is reduced compared to **(A)** due to slow residual inflow of blood substitute during polymerization. Recycled contrast agent after re-establishment of blood flow throughout the model is visible on **(C,D)**.

Figure 10 shows the progressive deflation and removal of the balloon and solidified photopolymerized hydrogel *in situ* in almost physiological hemodynamic conditions.

### Cell Viability of Precursor and Hydrogel

**FIGURE 11:**
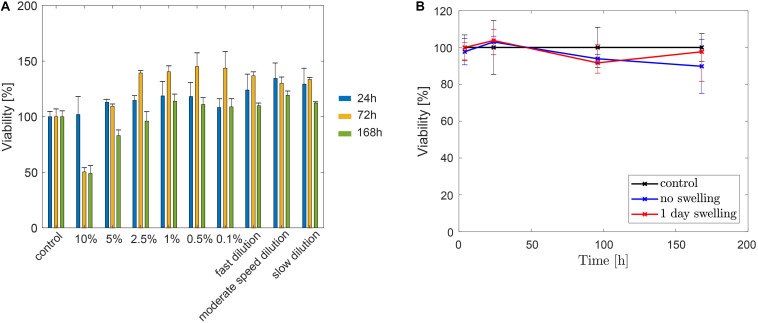
**(A)** HUVEC viability of different concentrations and dilutions of hydrogel precursors (*n* = 4). **(B)** HUVEC viability after 1 week of direct contact with PEGDMA hydrogels either immediately after polymerization or after 1 day of swelling in PBS following polymerization (*n* = 3).

As displayed in Figure 11A, the hydrogel precursor was diluted to different concentrations, ranging from 10 to 0.1%. The viability assay showed that a concentration of 10 vol% lead to 49.65 ± 3.77% cell death after 3 days of contact, which was non-significantly different after 1 week of contact. For concentration lower than 5 vol%, the viability increased after 3 days and a decrease was noticed after 1 week. However, this viability decrease was still acceptable, accordingly to ISO 10993-4 which requires a viability higher that 70%. Similarly, incubation of HUVEC cells with pure precursor during 10 min (representing the worst-case scenario with the most toxic concentration of leaked precursor in the parent artery during balloon inflation and *in situ* illumination) did not negatively impact viability, as shown for the serial dilutions at different speeds. This result suggests that longer contact time of low concentration hydrogel (<5 vol%) is highly unlikely to be toxic under realistic *in vivo* conditions.

Beyond a week of contact with HUVEC, polymerized hydrogels, directly after photopolymerization and after 1 day of swelling in PBS, exhibited a viability of 89.78 ± 14.65 and 97.65 ± 8.33, respectively, as shown in Figure 11B. Therefore, the hydrogel fulfilled the ISO 10993-4 requirements, implying no toxic effect of the hydrogel on HUVEC.

### Platelet Aggregation

**FIGURE 12:**
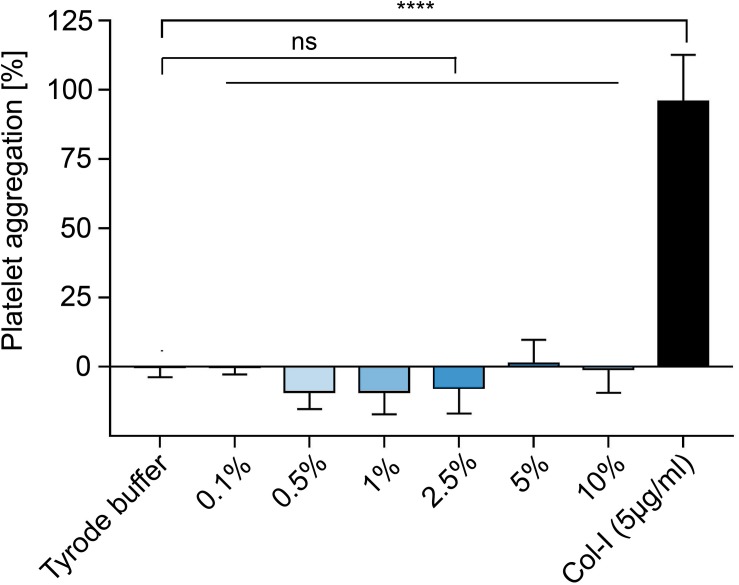
Platelet aggregation of different concentrations of hydrogel precursors (*n* = 3).

Platelet aggregation was measured for different concentrations of precursor, ranging from 0.1 to 10 vol% and compared with Tyrode buffer as negative control and Collagen I and PPP, as positive controls, as shown in Figure 12. The assay indicated the aggregation of the precursor at different concentrations are not significantly different from the negative control, where the platelets did not aggregate. The negative values of aggregation might be explained by the sampling variations (*n* = 3 donors, 3 samples/donor). Therefore, for concentration lower than 10 vol%, the precursor seems not to induce any platelet aggregation.

## Discussion

The present study is, to the best of our knowledge, the first to demonstrate the feasibility of *in situ* implementation using the same microcatheters as in clinical practice, as well as photoactivation and polymerization of hydrogels to treat IAs.

A major strength of our work is the development of a low viscosity precursor injectable through the same microcatheters as those used for coiling, i.e., with an inner diameter of 430 μm (1.7 French outer diameter). In comparison, the injectability of precursors until now was feasible through 4–6 French-catheters (1.33–2 mm outer diameter) (Takao et al., 2006; Ishikawa et al., 2012; Bearat et al., 2013; Weng et al., 2013; Avery et al., 2016; Shi et al., 2016). A pH-sensitive hydrogel was injected through a 2 French-catheter (490 μm inner diameter) (Nguyen et al., 2016), but so far we found no evidence of a similar or alternative hydrogel injectable through a 1.7 French-microcatheter or smaller. The viscosity in our hydrogel precursor was most affected by the iodinated contrast agent rather than the PEGDMA monomer. By using other clinically approved but less viscous contrast agents, it might be possible to further reduce resistance to injection in small microcatheters. Accupaque300 (GE Healthcare) or Visipaque270 (Nycomed), notably, have a viscosity at 20°C of 11.6 and 11.3 mPa.s, respectively, corresponding to a drop of almost 40%.

Radiopacity of the hydrogel is indispensable for aneurysm embolization. In this study, the addition of 50 wt% iodinated contrast agent in the composition allowed both precursor and hydrogel to be radiopaque. It has been shown that hydrogel radiopacity remains acceptable under fluoroscopy with 20–25 wt% iodinated contrast medium (Takao et al., 2006; Barnett et al., 2009; Fatimi et al., 2011; Nguyen et al., 2016). In other words, we could further decrease the radiopaque agent content in our formulation and hence viscosity, without significantly altering the fluoroscopic visibility of the liquid precursor. Radiopaque nanoparticles might also improve injectability to an even greater extent (Tian et al., 2018) and allow a higher percentage of polymer, thereby potentially shortening the photosolidification process to a minute or less. It is known that iodinated contrast agents are subject to leak to the surrounding environment (Lei et al., 2016), as it was demonstrated for our hydrogel over the following days due to the immersion process (Figure 6B). In contrast to coiling or clipping (Hänsel et al., 2018) or use of EVOH agents (Saatci et al., 2003) where artifacts are usually observed after treatment, the fast leakage of iodine has the potential to increase the quality of follow up imaging on Computed Tomography (CT) or Magnetic Resonance Imaging (MRI). The intra-aneurysmal content might become easier to evaluate in terms of recurrence without the need for follow up digital subtraction angiography (DSA), which requires arterial catheterization and still carries a small but relevant risk of iatrogenic ischemic stroke (Connors et al., 2005).

A further novelty of our approach is the use of a new type of water-soluble photoinitiator that is activated by visible light and allows the hydrogel to completely photopolymerize within 8 min. For biomedical applications, Irgacure2959 remains the most widely used water-soluble photoinitiator. Its major drawbacks are longer polymerization times, approximatively 25 min for 6 kDa PEGDMA hydrogels (Khoushabi et al., 2015), and the need for ultraviolet light, which may damage the DNA of the encapsulated cells or host tissues (Sinha and Häder, 2002; Fedorovich et al., 2009). Other water-soluble photoinitiators sensitive to visible light, such as lithium phenyl2,4,6-trimethylbenzoylphosphinate (LAP), 2,2′-azobis[2- methyl-*n*-(2-hydroxyethyl)propionamide] (VA-086) or eosin, used in tissue engineering (Wang Z. et al., 2018) might also be of interest. Intralipids were added to the composition for their intrinsic scattering properties. The impact of these particles were not significantly noticed in this test. However, it should be noted that the photorheology bench test we performed was on thin samples (300 μm thickness) under homogeneous illumination using 405 nm light over the whole surface of the sample. The effect of light scattering particles becomes significant when the sample is polymerized in depth and when light is not homogeneously distributed (Schmocker et al., 2014). Therefore, the small decrease of photopolymerization time might be explained by the scattering particles which help illuminate the sample in a homogenous manner. Therefore, we hypothesized that adding intralipids would enhance light distribution and photoactivation in more complex environments such as a human brain aneurysm, although this did not affect photopolymerization time in the test. In addition, the acceleration of the curing could also explain the reduction of the G’ modulus, since it might cause porosity in the hydrogel network and therefore lower mechanical properties.

A handful of studies have focused on the embolization of IAs using chemical and physical cross-linked hydrogels (Takao et al., 2006; Bearat et al., 2013; Weng et al., 2013), usually limited by premature gelation or later dilution. Takao et al. (2006) managed to embolize a wide-necked lateral wall aneurysm using a thermo-reversible hydrogel but with the drawback of solidifying into the microcatheter during delivery, rendering their applicability in humans unlikely. Consequently, a further benefit of the photopolymerizable hydrogel we have developed is the controllability of its solidification. Indeed, photoactivation can be triggered precisely by the operator by selectively switching the illumination module on. Moreover, it is theoretically possible to implement a feedback loop coupled to sound to monitor the solidification process by using light of a different wavelength through the same optical fiber (Schmocker et al., 2015).

The relevance of silicone aneurysms models for *in vitro* studies to test new devices, such as coils, stents or embolic agents was previously confirmed in multiple studies (Paramasivam et al., 2014). Similar *in vitro* pulsatile flow setups for aneurysm embolization (ranging from 100 to 300 ml/min) were developed in a few studies (Ishikawa et al., 2012; Bearat et al., 2013). A patient-specific carotid ophthalmic aneurysm silicone model enabled us to assess real-life navigation of the light-conducting microcatheter prototype, despite limitations such as the absence of endothelial lining or natural elastic and lubricity conditions. Nonetheless, microcatheterization of the aneurysm and visible light emission *in situ* was feasible in physiologically approaching hemodynamic conditions. Although a similar optical system was developed by Barnett et al. (2009) using a commercially available Excimer laser catheter sheath attached to a UV connection box for aortic and saccular aneurysm treatment, its minimal diameter of 0.9 mm limits its use for IAs, not to mention the danger of UV light previously discussed. Pushing an optical fiber through the inner lumen of a 430 μm microcatheter proved to be extremely time consuming in our experience and almost impossible in case of curvy or loopy anatomy. The risk of breaking the optic fiber was high, as well as the risk of vessel wall perforation related to the absence of radiopaque marker at the distal tip. The heat shrink tube ensured mechanical protection of the fibers but increased the bulkiness of the microcatheter. Braiding the optical fibers directly in the plastic wall of the microcatheter would be an elegant way to ensure mechanical protection without increasing the overall diameter.

A limitation of our implementation system is that it is difficult to rinse the blood out of the aneurysm entirely with the precursor and to keep the latter trapped inside to fill the whole volume before photoactivation. It is also challenging to avoid blood contamination from residual inflow during balloon inflation that could hinder the polymerization process. We tried to solve this issue by inflating the balloon in a submaximal fashion so that the precursor could be injected at a rate higher than the residual blood inflow until it leaked out into the parent artery, at which point the balloon was inflated to obtain complete sealing of the neck. It remains to be shown whether this technical feature is applicable in a real clinical situation.

Since leakage into the parent artery is virtually inevitable and should be expected, biocompatibility of the liquid precursor is essential. Compared to precipitating EVOH embolic agents containing potentially toxic DMSO (Santos et al., 2003) or to rapidly polymerizing substances such acrylic glues (Suh et al., 2003), the photoactive hydrogel precursor we have developed consists of clinically approved solvents only, can be selectively polymerized on demand with visible light and demonstrates no toxicity for endothelial cells in both its liquid and solid states. Contrary to chemical, temperature- or pH-sensitive hydrogel precursors that have a high risk of polymerizing in the parent artery in case of leakage, our preliminary tests indicate that our light-sensitive precursor should be harmless if injected into the blood stream and should not induce any platelet aggregation. Nevertheless, cytotoxicity and thrombogenicity have to be further studied *in vitro* and *in vivo*, which we will test systemically in rats before moving to *in situ* photopolymerization in an elastase-aneurysm model in rabbits (Brinjikji et al., 2016).

## Conclusion

Our study reveals the potential of photopolymerizable hydrogels for IA treatment. The developed hydrogel was shown to have a low viscosity enabling injection through 430 μm microcatheters, to solidify within less than 10 min using visible light and to be radiopaque under fluoroscopy. The proof-of-concept of *in situ* implementation of the hydrogel was validated in a realistic flow model of a human brain aneurysm replica, using a customized light-conducting microcatheter. In addition, the precursor and hydrogel were proved to be biocompatible.

While this new type of liquid embolic agent has the potential to overcome the limitations of current endovascular implants, further biomechanical characterization, as well as *in vivo* studies are needed to confirm these preliminary results and to evaluate other key features, such as clinical safety, thrombogenicity, tissue adherence, endothelialization and long-term durability.

## Data Availability Statement

The datasets generated for this study are available on request to the corresponding author.

## Author Contributions

OP developed the hydrogels, performed the rheology and photorheology measurements and the cell viability assay. AS designed the light-conducting microcatheter and the illumination module. RC synthesized the chemical components of the hydrogels. OP, AS, and PM designed the *in vitro* setup and performed the feasibility test as well as the radiopacity evaluation. PM and DP supervised the project and share equal senior contributions. OP wrote the manuscript and prepared the figures. AS, PM, and DP reviewed the manuscript. All authors commented on the manuscript.

## Conflict of Interest

AS is a shareholder at Lumendo SA to which the intellectual property was licensed. The authors declare that the research was conducted in the absence of any commercial or financial relationships that could be construed as a potential conflict of interest.

## References

[B1] Abdihalim M. Watanabe M. Chaudhry S. A. Jagadeesan B. Suri M. F. K. Qureshi A. I. (2014). Are coil compaction and aneurysmal growth two distinct etiologies leading to recurrence following endovascular treatment of intracranial aneurysm? *J. Neuroimaging* 24 171–175. 10.1111/j.1552-6569.2012.00786.x 23317437

[B2] Avery R. K. Albadawi H. Akbari M. Zhang Y. S. Duggan M. J. Sahani D. V. (2016). An injectable shear-thinning biomaterial for endovascular embolization. *Sci. Transl. Med.* 8:365ra156. 10.1126/scitranslmed.aah5533 27856795

[B3] Barnett P. Hughes A. H. Lin S. Arepally A. Gailloud P. H. (2009). In vitro assessment of Embogel and ultragel radiopaque hydrogels for the endovascular treatment of aneurysms. *J. Vasc. Interv. Radiol.* 20 507–512. 10.1016/j.jvir.2009.01.005 19328428

[B4] Bearat H. H. Preul M. C. Vernon B. L. (2013). Cytotoxicity, in vitro models and preliminary in vivo study of dual physical and chemical gels for endovascular embolization of cerebral aneurysms. *J. Biomed. Mater. Res. Part A* 101A 2515–2525. 10.1002/jbm.a.34554 23359550

[B5] Bonneville F. Sourour N. Biondi A. (2006). Intracranial aneurysms: an overview. *Neuroimaging Clin. N. Am.* 16 371–382. 16935705 10.1016/j.nic.2006.05.001

[B6] Brassel F. Meila D. (2015). Evolution of embolic agents in interventional neuroradiology. *Clin. Neuroradiol.* 25 333–339. 10.1007/s00062-015-0419-6 26084977

[B7] Brinjikji W. Ding Y. H. Kallmes D. F. Kadirvel R. (2016). From bench to bedside: utility of the rabbit elastase aneurysm model in preclinical studies of intracranial aneurysm treatment. *J. Neurointerv. Surg.* 8 521–525. 10.1136/neurintsurg-2015-011704 25904642 PMC4932861

[B8] Burke G. Barron V. Geever T. Geever L. Devine D. M. Higginbotham C. L. (2019). Evaluation of the materials properties, stability and cell response of a range of PEGDMA hydrogels for tissue engineering applications. *J. Mech. Behav. Biomed. Mater.* 99 1–10. 10.1016/j.jmbbm.2019.07.003 31319331

[B9] Connors J. Sacks D. Furlan A. J. Selman W. R. Russell E. J. Stieg P. E. (2005). Training, competency, and credentialing standards for diagnostic cervicocerebral angiography, carotid stenting, and cerebrovascular intervention: a joint statement from the american academy of neurology, the american association of neurological surgeons. *Neurology* 64 190–198. 10.1212/01.wnl.0000148958.34025.09 15668413

[B10] Corrigan N. Yeow J. Judzewitsch P. Xu J. Boyer C. (2019). Seeing the light: advancing materials chemistry through photopolymerization. *Angew. Chemie Int. Ed.* 58 5170–5189. 10.1002/anie.201805473 30066456

[B11] Dimatteo R. Darling N. J. Segura T. (2018). In situ forming injectable hydrogels for drug delivery and wound repair. *Adv. Drug Deliv. Rev.* 127 167–184. 10.1016/j.addr.2018.03.007 29567395 PMC6003852

[B12] Doran S. Taskin O. S. Tasdelen M. A. Yaðci Y. (2015). “Controlled photopolymerization and novel architectures,”in *Dyes and Chromophores in Polymer Science.* eds Lalevée J. Fouassier J. P. (Hoboken, NJ: John Wiley & Sons, Inc), 81–121. 10.1002/9781119006671.ch3

[B13] Enzmann R. Ross M. R. Marks M. P. (1994). Blood flow in major cerebral arteries measured by phase-contrast cine MR. *Am. J. Neuroradiol.* 15 123–129. 8141043 PMC8332086

[B14] Fatimi J. M. Coutu G. C. Lerouge S. (2011). Rheological studies of an injectable radiopaque hydrogel for embolization of abdominal aortic aneurysms. *Adv. Mater. Res.* 409 129–135. 10.4028/www.scientific.net/amr.409.129

[B15] Fedorovich N. E. Oudshoorn M. H. van Geemen D. Hennink W. E. Alblas J. Dhert W. J. A. (2009). The effect of photopolymerization on stem cells embedded in hydrogels. *Biomaterials* 30 344–353. 10.1016/j.biomaterials.2008.09.037 18930540

[B16] Hänsel N. H. Schubert G. A. Scholz B. Nikoubashman O. Othman A. E. Wiesmann M. (2018). Implant-specific follow-up imaging of treated intracranial aneurysms: TOF-MRA vs. metal artifact reduced intravenous flat panel computed tomography angiography (FPCTA). *Clin. Radiol.* 73 218.e9–218.e15. 10.1016/j.crad.2017.07.011 28811040

[B17] Hulsbergen F. C. Mirzaei L. van der Boog L. Smith A. T. J. Muskens T. R. Broekman I. S. (2019). Long-term durability of open surgical versus endovascular repair of intracranial aneurysms: a systematic review and meta-analysis. *World Neurosurg.* 132 e820–e833. 10.1016/j.wneu.2019.08.002 31419590

[B18] Ishikawa A. Nakayama Y. Kambe N. (2012). In vitro study of photocurable embolization agent for cerebral aneurysms. *J. Biotechnol. Biomater.* 2:128.

[B19] Khoushabi A. Schmocker A. Pioletti D. P. Moser C. (2015). Photo-polymerization, swelling and mechanical properties of cellulose fibre reinforced poly(ethylene glycol) hydrogels. *Compos. Sci. Technol.* 119 93–99. 10.1016/j.compscitech.2015.10.002

[B20] Kilani M. S. Izaaryene J. Cohen F. Varoquaux A. Gaubert J. Y. Louis G. (2015). Ethylene vinyl alcohol copolymer (Onyx^®^) in peripheral interventional radiology?: indications, advantages and limitations. *Diagn. Interv. Imaging* 96 319–326. 10.1016/j.diii.2014.11.030 25704146

[B21] Lei K. Ma Q. Yu L. Ding J. (2016). Functional biomedical hydrogels for in vivo imaging. *J. Mater. Chem. B* 4 7793–7812. 10.1039/c6tb02019d 32263771

[B22] Li S. Dong S. Xu W. Tu S. Yan L. Zhao C. (2018). Antibacterial hydrogels. *Adv. Sci.* 5:1700527. 10.1002/advs.201700527 PMC5980143 29876202

[B23] Lim W. L. Chew Y. T. Chew T. C. Low H. T. (2001). Pulsatile flow studies of a porcine bioprosthetic aortic valve in vitro: PIV measurements and shear-induced blood damage. *J. Biomech.* 34 1417–1427. 10.1016/s0021-9290(01)00132-4 11672716

[B24] Lima M. A. Bragina M. E. Burri O. Bortoli Chapalay J. Costa-Fraga F. P. Chambon M. (2019). An optimized and validated 384-well plate assay to test platelet function in a high-throughput screening format. *Platelets* 30 563–571. 10.1080/09537104.2018.1514106 30183501

[B25] Lin-Gibson S. Bencherif S. Cooper J. A. Wetzel S. J. Antonucci J. M. Vogel B. M. (2004). Synthesis and characterization of PEG dimethacrylates and their hydrogels. *Biomacromolecules* 5 1280–1287. 10.1021/bm0498777 15244441

[B26] Marbacher S. Niemelä M. Hernesniemi J. Frösén J. (2019). Recurrence of endovascularly and microsurgically treated intracranial aneurysms-review of the putative role of aneurysm wall biology. *Neurosurg. Rev.* 42 49–58. 10.1007/s10143-017-0892-2 28819834

[B27] Medical Advisory Secretariat (2006). Coil embolization for intracranial aneurysms: an evidence-based analysis. *Ont. Health Technol. Assess. Ser.* 6 1–114. 23074479 PMC3379525

[B28] Molyneux J. Birks J. Clarke A. Sneade M. Kerr R. S. C. (2015). The durability of endovascular coiling versus neurosurgical clipping of ruptured cerebral aneurysms: 18 year follow-up of the UK cohort of the International Subarachnoid Aneurysm Trial (ISAT). *Lancet* 385 691–697. 10.1016/S0140-6736(14)60975-2 25465111 PMC4356153

[B29] Netlyukh M. Shevaga V. M. Yakovenko L. M. Payenok A. V. Salo V. M. Kobyletskiy O. J. (2015). Invasive intracranial arterial pressure monitoring during endovascular cerebral aneurysms embolization for cerebral perfusion evaluation. *Acta Neurochir. Suppl.* 120 177–181. 10.1007/978-3-319-04981-6_30 25366620

[B30] Nguyen Q. V. Lee M. S. Lym J. S. Il Kim Y. Jae H. J. Lee D. S. (2016). pH-Sensitive sulfamethazine-based hydrogels as potential embolic agents for transcatheter vascular embolization. *J. Mater. Chem. B* 4 6524–6533. 10.1039/c6tb01690a 32263697

[B31] Onyx^®^ Liquid Embolic System Onyx^®^ Hd-500 (2007). Available online at: https://www.accessdata.fda.gov/cdrh_docs/pdf6/H060003C.pdf (accessed July 5, 2017).

[B32] Paramasivam S. Baltsavias G. Psatha E. Matis G. Valavanis A. (2014). Silicone models as basic training and research aid in endovascular neurointervention—a single-center experience and review of the literature. *Neurosurg. Rev.* 37 331–337. 10.1007/s10143-014-0518-x 24463914

[B33] Ravindran K. Salem M. M. Alturki A. Y. Thomas A. J. Ogilvy C. S. Moore J. M. (2019). Endothelialization following flow diversion for intracranial aneurysms: a systematic review. *Am. J. Neuroradiol.* 40 295–301. 10.3174/ajnr.A5955 30679207 PMC7028638

[B34] Saatci H. S. Cekirge E. F. Ciceri M. Mawad M. E. Pamuk A. G. Besim A. (2003). CT and MR imaging findings and their implications in the follow-up of patients with intracranial aneurysms treated with endosaccular occlusion with onyx. *AJNR Am. J.* 24 567–578. 12695183 PMC8148677

[B35] Santos N. C. Figueira-Coelho J. Martins-Silva J. Saldanha C. (2003). Multidisciplinary utilization of dimethyl sulfoxide: pharmacological, cellular, and molecular aspects. *Biochem. Pharmacol.* 65 1035–1041. 10.1016/s0006-2952(03)00002-9 12663039

[B36] Schmocker A. Khoushabi C. Schizas Bourban P.-E. Pioletti D. P. Moser C. (2014). Photopolymerizable hydrogels for implants: monte-carlo modeling and experimental in vitro validation. *J. Biomed. Opt.* 19:35004. 10.1117/1.JBO.19.3.035004 24615642

[B37] Schmocker C. Schizas P. B. Pioletti D. P. Moser C. (2015). Miniature probe for the delivery and monitoring of a photopolymerizable material. *J. Biomed. Opt.* 20:127001. 10.1117/1.JBO.20.12.127001 26662066

[B38] Shi X. Gao H. Dai F. Feng X. Liu W. (2016). A thermoresponsive supramolecular copolymer hydrogel for the embolization of kidney arteries. *Biomater. Sci.* 4 1673–1681. 10.1039/c6bm00597g 27709136

[B39] Sinha R. P. Häder D. P. (2002). UV-induced DNA damage and repair: a review. *Photochem. Photobiol. Sci.* 1 225–236. 10.1055/s-0028-1109836 12661961

[B40] Smith T. R. Cote D. J. Dasenbrock H. H. Hamade Y. J. Zammar S. G. El Tecle NE, et al. (2015). Comparison of the efficacy and safety of endovascular coiling versus microsurgical clipping for unruptured middle cerebral artery aneurysms: a systematic review and meta-analysis. *World Neurosurg.* 84 942–953. 10.1016/j.wneu.2015.05.073 26093360

[B41] Spiotta M. Bhalla T. Hussain M. S. Sivapatham T. Batra A. Hui F. (2011). An analysis of inflation times during balloon-assisted aneurysm coil embolization and ischemic complications. *Stroke* 42 1051–1055. 10.1161/STROKEAHA.110.602276 21311066

[B42] Suh C. Kim K. S. Lim S. M. Shi H. B. Choi C. G. Lee H. K. (2003). Technical feasibility of embolizing aneurysms with glue (n-butyl 2-cyanoacrylate): experimental study in rabbits. *Am. J. Neuroradiol.* 24 1532–1539. 13679265 PMC7973981

[B43] Takao H. Murayama Y. Saguchi T. Ishibashi T. Ebara M. Irie K. (2006). Endovascular treatment of experimental cerebral aneurysms using thermoreversible liquid embolic agents. *Interv. Neuroradiol.* 12 154–157. 10.1177/15910199060120s126 20569622 PMC3387944

[B44] Takeda M. Pokorski Y. Sato Oyamada Y. Okada Y. (2015). Respiratory toxicity of dimethyl sulfoxide. *Adv. Exp. Med. Biol.* 885 89–96. 10.1007/5584_2015_187 26747070

[B45] Tian L. Lu L. Feng J. Melancon M. P. (2018). Radiopaque nano and polymeric materials for atherosclerosis imaging, embolization and other catheterization procedures. *Acta Pharm. Sin. B* 8 360–370. 10.1016/j.apsb.2018.03.002 29881675 PMC5990339

[B46] Tulamo R. Frosen J. Hernesniemi J. Niemela M. (2010). Inflammatory changes in the aneurysm wall: a review. *J. Neurointerv. Surg.* 2 120–130. 10.1136/jnis.2009.002055 21990591

[B47] Vollherbst F. Sommer C. M. Ulfert C. Pfaff J. Bendszus M. Möhlenbruch M. A. (2017). Liquid embolic agents for endovascular embolization: evaluation of an established (Onyx) and a novel (PHIL) embolic agent in an in vitro AVM model. *Am. J. Neuroradiol.* 38 1377–1382. 10.3174/ajnr.A5203 28522669 PMC7959908

[B48] Walcott P. Gerrard J. L. Nogueira R. G. Nahed B. V. Terry A. R. Ogilvy C. S. (2011). Microsurgical retrieval of an endovascular microcatheter trapped during Onyx embolization of a cerebral arteriovenous malformation. *J. Neurointerv. Surg.* 3 77–79. 10.1136/jnis.2010.002733 21990795

[B49] Wang J. Stanic S. Altun A. A. Schwentenwein M. Dietliker K. Jin L. (2018). A highly efficient waterborne photoinitiator for visible-light-induced three-dimensional printing of hydrogels. *Chem. Commun.* 54 920–923. 10.1039/c7cc09313f 29318224

[B50] Wang Z. Kumar H. Tian Z. Jin X. Holzman J. F. Menard F. (2018). Visible light photoinitiation of cell-adhesive gelatin methacryloyl hydrogels for stereolithography 3D bioprinting. *ACS Appl. Mater. Interf.* 10 26859–26869. 10.1021/acsami.8b06607 30024722

[B51] Weng L. Rostambeigi N. Zantek N. D. Rostamzadeh P. Bravo M. Carey J. (2013). An in situ forming biodegradable hydrogel-based embolic agent for interventional therapies. *Acta Biomater.* 9 8182–8191. 10.1016/j.actbio.2013.06.020 23791672

[B52] Zhang Y. Yu J. Ren K. Zuo J. Ding J. Chen X. (2019). Thermosensitive hydrogels as scaffolds for cartilage tissue engineering. *Biomacromolecules* 20 1478–1492. 10.1021/acs.biomac.9b00043 30843390

[B53] Zhao H. Shen J. Duan P. Xia X. Chen R. Jin B. (2013). Temperature-sensitive poly(N -Isopropylacrylamide-Co-butyl methylacrylate) nanogel as an embolic agent: distribution, durability of vascular occlusion, and inflammatory reactions in the renal artery of rabbits. *Am. J. Neuroradiol.* 34 169–176. 10.3174/ajnr.A3177 22859278 PMC7966348

[B54] Zhou F. An Q. Chen L. Wen Y. Fang F. Zhu W. (2016). Novel hydrogel material as a potential embolic agent in embolization treatments. *Sci. Rep.* 6:32145. 10.1038/srep32145 27561915 PMC4999878

