# Supplementary figures and images for: In vitro Implementation of Photopolymerizable Hydrogels as a Potential Treatment of Intracranial Aneurysms
Source: Front Bioeng Biotechnol. 2020 Apr 3;8:261. doi: 10.3389/fbioe.2020.00261 (PMC7146053; doi:10.3389/fbioe.2020.00261)

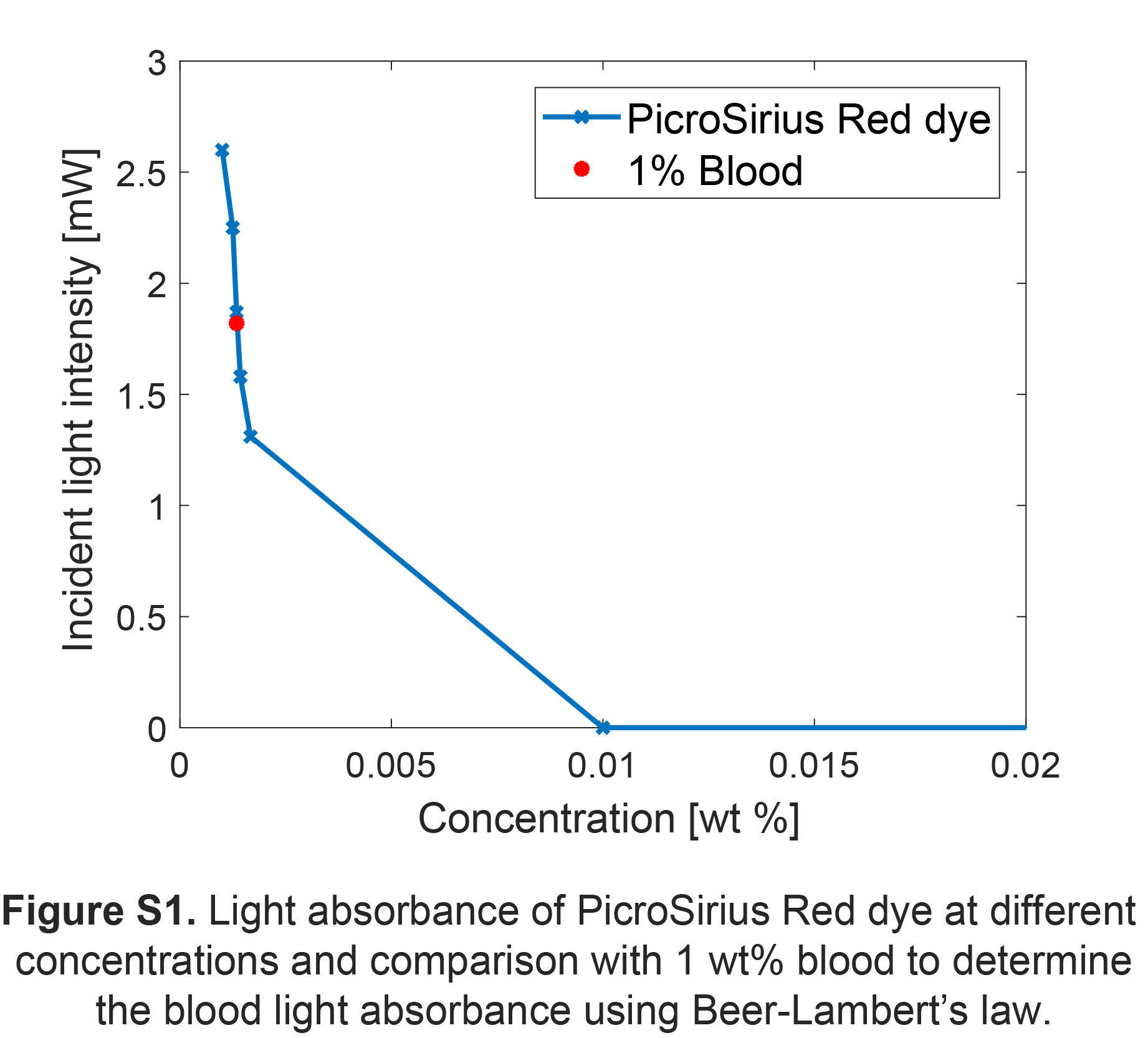

Supplement: Supplementary file 1 [file Image_1.JPEG]

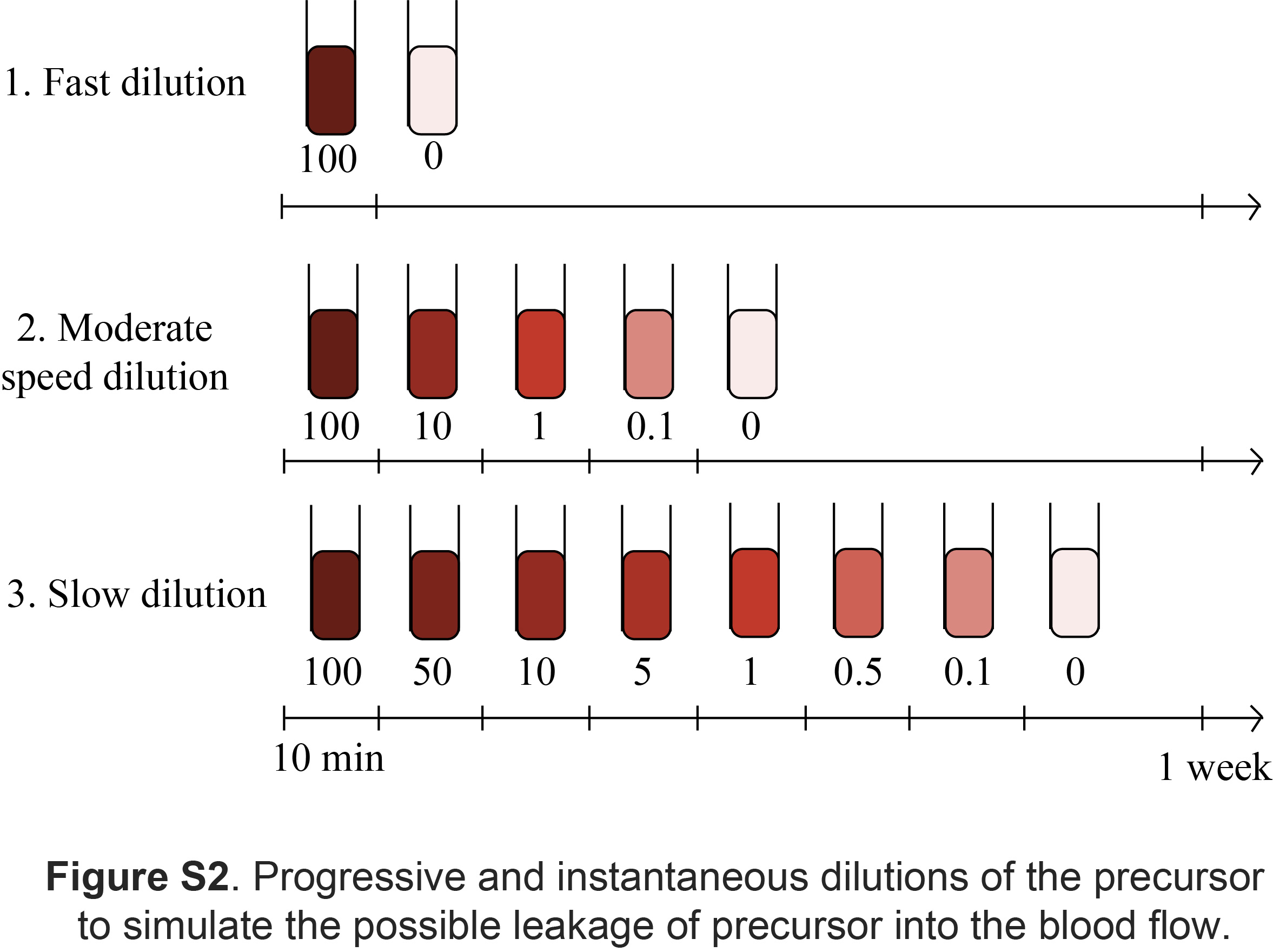

Supplement: Supplementary file 2 [file Image_2.JPEG]
